# Muscle structure predictors of vertical jump performance in elite male volleyball players: a cross-sectional study based on ultrasonography

**DOI:** 10.3389/fphys.2024.1427748

**Published:** 2024-07-30

**Authors:** WeiDong Jiang, Chao Chen, Yilin Xu

**Affiliations:** ^1^ School of Physical Education and Training, Shanghai University of Sport, Shanghai, China; ^2^ School of Competitive Sports, Shanghai University of Sport, Shanghai, China; ^3^ Sports Biomechanics Laboratory, Jiangsu Research Institute of Sports Science, Nanjing, Jiangsu, China

**Keywords:** ultrasonography, anatomical cross-sectional area, fascicle length, spike jump, performance

## Abstract

**Objective:**

The objective of this investigation is to examine the contribution of key muscle groups in the lower limbs to vertical jumping performance in elite male volleyball players. Specifically, the study focuses on the rectus femoris (RF), vastus lateralis (VL), and lateral gastrocnemius (LG), as well as exploring differences between attack jump and other vertical jump types.

**Methods:**

To achieve this, we employed B-mode ultrasound to evaluate the anatomical cross-sectional area (ACSA), muscle thickness (MT), pennation angle (PA), and fascicle length (FL) of the RF, VL, and LG in the participants. Fifteen elite male volleyball players were recruited as participants for this study. Jump heights were measured for four types of vertical jumps: attack jump (AJ), countermovement jump (CMJ), squat jump (SJ), and drop jump (DJ). We conducted regression analyses to assess whether the previously mentioned muscle structures could predict jump performance.

**Results:**

Our findings reveal that the muscle structure of the RF does not exhibit any significant correlation with the height of any jump. However, VL-ACSA displays a significant and the most potent predictive effect on jump height for all four jump types (AJ: *R* ^*2*^ = 0.32, *p* = 0.001; CMJ: *R* ^*2*^ = 0.37, *p* = 0.005; SJ: *R* ^*2*^ = 0.52, *p* = 0.001; DJ: *R* ^*2*^ = 0.25, *p* = 0.021). Conversely, LG-FL only demonstrates a significant and stronger predictive effect on AJ jump height (*R* ^*2*^ = 0.18, *p* = 0.009). Combining VL-ACSA, LG-FL, and training age through multiple linear regression analysis resulted in a highly significant model for predicting AJ jump height (*F* = 13.86, *R* ^*2*^ = 0.73). Moreover, the model incorporating VL-ACSA and training age is also important for predicting CMJ, SJ, and DJ jump heights (*F* = 8.41, *R* ^*2*^ = 0.51; *F* = 13.14, *R* ^*2*^ = 0.63; *F* = 5.95, *R* ^*2*^ = 0.41; respectively).

**Conclusion:**

The muscle structure indicators in the lower limbs significantly predict jump performance among elite male volleyball players. However, different jump types are influenced by distinct indicators, particularly in the case of AJ, which is associated with LG-FL. This suggests that enhancing LG-FL may positively impact AJ ability, thereby emphasizing the importance of specificity in training. To optimize specialized jump performance in volleyball players, practitioners are advised to assess VL-ACSA and LG-FL and incorporate step-up and eccentric strength training targeting the calf muscles to yield considerable benefits.

## Introduction

Volleyball is a sport that requires overcoming body weight and completing various explosive movements in a short period of time. The special actions in volleyball such as jumping, landing, blocking, and spiking require fast explosive movements, placing high demands on the athletes’ lower limb neuromusculoskeletal system, making vertical jumping ability a key factor for success (Sheppard et al., 2009; Xu et al., 2022; Xu et al., 2023). Among the various types of vertical jumps, attack jumping (AJ) is especially important for improving volleyball performance (Marcelino and Milanovic, 2000; Fuchs et al., 2021). The height reached during attacking also influences the outcomes of both men’s and women’s volleyball matches (Berriel et al., 2021).

Muscle structure refers to how muscle fibers are aligned for the direction of force, forming the macroscopic configuration of muscle fibers (Maden-Wilkinson et al., 2021). Ultrasound (US) scanning is an imaging technique that is efficient, accessible, swift, and cost-effective and has been widely used to evaluate muscle structure (Lichtwark et al., 2007; Aggeloussis et al., 2010; Lieber and Ward, 2011). Some validation studies have compared ultrasound measurements with reference standards such as magnetic resonance imaging (MRI) or cadaveric dissection (Ema et al., 2013; Franchi et al., 2018). These studies not only demonstrate a high level of agreement between ultrasound and MRI measurements in various muscle structural parameters but also indicate good reliability and validity of ultrasound measurements in assessing muscle structure (Betz et al., 2021; Narici, 1999). Furthermore, previous research has confirmed the reliability and validity of ultrasound measurements for assessing the muscle structure of vastus lateralis (VL) (Raj et al., 2012; Betz et al., 2021), rectus femoris (RF) (Lima et al., 2012; Ema et al., 2013), and lateral gastrocnemius (LG) (Kwah et al., 2013; May et al., 2021). The technique provides reliable measurements of different aspects of muscle architecture, such as muscle anatomical cross-sectional area (ACSA), muscle thickness (MT), fascicle length (FL), and pennation angle (PA). Moreover, ultrasound imaging can assess these features of muscle structure (Kwah et al., 2013) (with an average intra-class correlation coefficient above 0.7) in both the contracted and relaxed states of muscle, confirming its effectiveness.

Muscle architecture is one of the fundamental factors that affect muscular force and explosiveness (Suchomel et al., 2016; Franchi et al., 2018; Maden-Wilkinson et al., 2021). For athletes, having strong and explosive muscles is essential for high vertical jumping ability and overall athletic performance (Secomb et al., 2015). Long-term physical training can lead to changes in muscle ACSA, MT, FL, and PA, thereby improving muscle strength and explosiveness levels (Narici et al., 1996; Blazevich, 2006; Pareja-Blanco et al., 2017). This training method has a significant impact on performance in activities such as jumping, sprinting, and change of direction, and it also plays an important role in specialized sports skills and performance (Suchomel et al., 2018; Suchomel et al., 2016). Previous studies have found significant differences in lower limb muscle architecture between trained athletes and untrained individuals, such as variations in fascicle length, pennation angle, and thickness (Maughan et al., 2004; Alegre et al., 2005). Elite athletes usually have better muscle force, explosiveness, and architecture than amateur athletes, which may explain their outstanding performance (Methenitis et al., 2016; Sarto et al., 2021). Lower limb architecture plays a key role in influencing lower limb explosiveness and jumping ability. Optimizing lower limb muscle architecture can potentially increase vertical jumping height (Secomb et al., 2015). Therefore, changes in muscle thickness (Lichtwark et al., 2007; Aggeloussis et al., 2010; Earp et al., 2010), fascicle length (Aggeloussis et al., 2010; Earp et al., 2010), pennation angle (Lichtwark et al., 2007; Earp et al., 2010; Kwah et al., 2013), and anatomical cross-sectional area (Suchomel et al., 2018; Suchomel and Stone, 2017) in the lower limb may significantly affect volleyball attack jumping height (Blazevich et al., 2009). Moreover, different sports and athlete levels have unique body morphology and muscle architecture features, indicating sport-specific adaptations (Secomb et al., 2016; Zaras et al., 2022).

Previous studies suggest that RF, VL, and LG are representative muscles of the lower extremity that strongly correlate with the general vertical jumping ability (CMJ, SJ, DJ) (Earp et al., 2010; Secomb et al., 2015; Ruiz-Cardenas et al., 2018). Therefore, these muscular structures can indirectly reflect an athlete’s jumping aptitude, provide valuable information about their physical condition, and facilitate targeted improvement of lower extremity muscular structures to enhance jumping performance (Lees et al., 2004). However, due to the lack of evidence in this domain, it is unclear which muscular structures of the lower extremity reflect the specialized jumping ability of elite volleyball players.

Standardized jump tests measure general vertical jump ability, but they may not reflect how athletes perform in real competition or training situations. Different events require different technical jump forms, which affect the effective competition performance (Jidovtseff et al., 2014). For example, in volleyball, the attack jump is a key skill that determines the attack’s success. Therefore, this study aimed to investigate how the lower limb muscle structure of elite male volleyball players relates to their attack jump height. We also wanted to find out which lower limb muscle structure can predict the attack jump height, and how it differs from the one that influences the general vertical jump height. We hypothesized that the muscular architecture of RF, VL, and LG associated with general vertical jump performance can also predict the attack jump height, but with different specific muscular structure indices. Our results will help practitioners design targeted training plans to improve the attack jump ability of elite volleyball players and provide new insights and theoretical bases for further research.

## Materials and methods

### Experimental approach to the problem

This study adopted a mixed experimental design, incorporating block randomization, repeated measures, and regression analysis, to evaluate the impact of the muscle structure of RF, VL, and LG on the jump height of AJ, CMJ, SJ, and DJ. This design addresses multiple variables and their effects, controls for potential biases, and accounts for individual differences. By quantifying the specific contributions of different muscle structures to the heights of different types of jumps, this approach enhances the reliability of the results and strengthens the scientific rigor of the study. All participants first underwent a B-mode ultrasound test, followed by two or three trials of each type of jump test, with a 2–3 min interval between each jump. Each participant completed all tests in one session at a fixed time on the same day.

### Subjects

Fifteen elite male volleyball athletes participated in this study (Tables 1, 2), All subjects had at least two Chinese national competitions or world international competition experiences, and five of them were former members of the Chinese national team. Thus, all subjects participated in national competitions and were international players who had taken part in international competitions. All subjects underwent at least 1 month of pre-competition preparation before testing. This study only included those who had not suffered from serious skeletal muscle or joint diseases that could have affected the study results within 2 months before the testing. During the study, none of the players took performance-enhancing drugs.

**TABLE 1:** Basic information of the subjects (n = 15).

Variable	Mean	*SD*
Age (yr)	22.47	4.09
Height (cm)	195.4	7.3
Body mass (kg)	90.9	9.55
Years of professional training (yr)	7.13	3.64

**TABLE 2:** Mean ± *SD* of jumping heights among elite male volleyball players: AJ, CMJ, SJ, and DJ (n = 15).

Variable	Mean	*SD*
AJ (m)	0.55	0.07
CMJ (m)	0.42	0.06
SJ (m)	0.40	0.06
DJ (m)	0.55	0.07

AJ, attack jump; CMJ, counter movement jump; SJ, squat jump; DJ, drop jump.

All subjects were informed of the study’s purpose and process and understood the experimental intent. They voluntarily participated in this study and signed an informed consent form.

### Procedures

#### Body composition test

Athletes were advised to hydrate adequately before the test. Each participant underwent all tests in one session. Before warming up, the participants wore sportswear and were barefooted, and their height and weight were measured using an electronic scale (model INDEX S2, GARMIN company) and a height-measuring ruler.

#### B-mode ultrasound test

Musculoskeletal ultrasound testing is performed using a real-time B-mode ultrasound imaging system (LOGIQ 𝘦 NextGen Ultrasound, GE Healthcare, United States) with a linear probe (L4-12T-RS) ranging from 4.2 to 13.0 MHz. The extended field of view (EFOV) mode (LOGIQview) is used. This equipment and technique have been validated in ultrasound measurements of muscle structure (Noorkoiv N. et al., 2010; Noorkoiv M. et al., 2010; Kwah et al., 2013).

**FIGURE 1:**
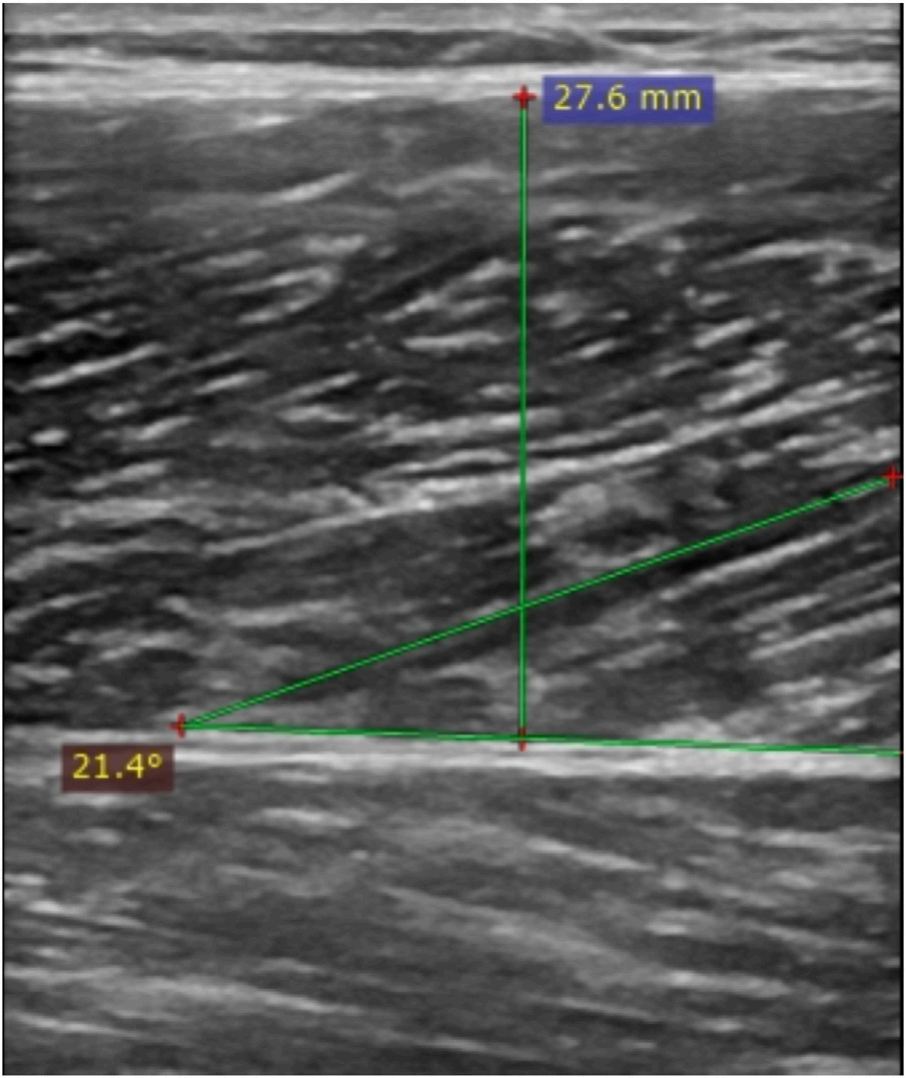
A typical musculoskeletal ultrasound image, demonstrating the thickness (MT) and pennation angle (PA) of the vertical muscle layers between the superficial fascia (SA) and deep fascia (DA) (indicated by the green lines).

**FIGURE 2:**
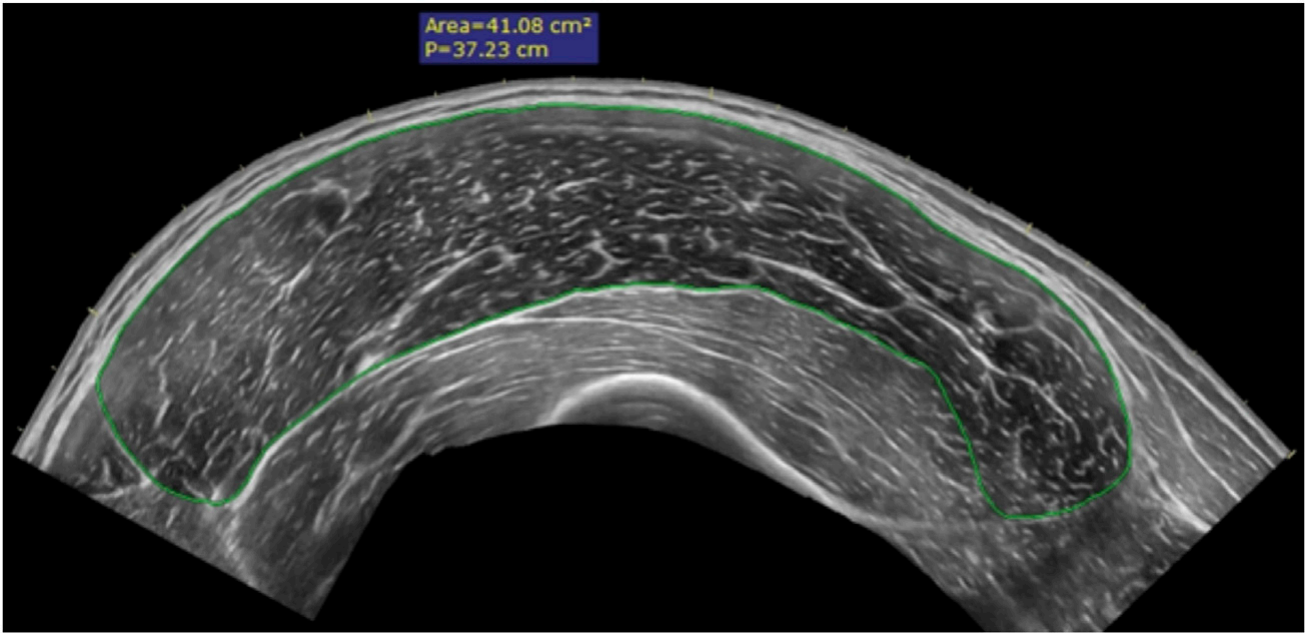
A typical panoramic ultrasound image is used to measure the anatomical cross-sectional area (ACSA) within the fascia of skeletal muscles, indicated by the green line.

The B-mode ultrasound assessment protocol is articulated as follows (Betz et al., 2021; Coratella et al., 2019; Ema et al., 2013; May et al., 2021): 1) Before the formal examination begins, the examiner explains the procedure and requirements of the ultrasound examination to the subjects, instructing them to wear shorts, remove their shoes, and assume a supine, lateral, or prone position on the examination table. The hip joint is extended, and the knee joint is nearly fully extended (170 of extension, complete extension of 180°) for measurement; 2) The examiner selects the RF measurement point at 50% distance between the center point of the patella and the anterior superior iliac spine, and marks the orientation line (Ema et al., 2013). The VL measurement point is selected at 50% distance between the greater tuberosity and the lateral condyle of the femur, and the orientation line is marked (Coratella et al., 2019). The LG measurement point is selected at the widest part of the muscle belly between the lateral condyle of the femur and the lateral malleolus (60%–70%), and the orientation line is marked (May et al., 2021); 3) The probe is oriented longitudinally (parallel to the femur) with the measurement point coinciding with the probe’s midpoint. A slight tilting motion is employed to procure standardized, lucid images for MT and PA measurements (Figure 1); 4) To quantify muscle ACSA, extended field of view (EFOV) software (LOGIQview, GE Healthcare GmbH) is utilized to generate a panoramic image of the skeletal muscle’s belly. The probe is positioned transversely (orthogonal to the femur and along the pre-marked linkage line), initiating from the muscle’s inner edge. Subsequently, the EFOV software is activated, and the probe is gradually moved along the line to the muscle’s outer edge, maintaining a constant velocity and minimal pressure (Figure 2); 5) While assessing the thigh with a probe, it is imperative to ensure ample water-soluble gel application between the probe and skin while preserving minimal pressure during scanning. A minimum of three distinct images are captured and archived at each marked point, under conditions of the participants in supine, lateral, or prone positions, with fully extended legs and relaxed muscles; 6) To ascertain that measurements are obtained under conditions of limb immobility, muscle relaxation, and normal joint alignment, foam pads are strategically placed under knee joints when supine, between ankle joints and knee joints during lateral positioning, and in front of ankle joints when prone, to alleviate muscle tension and maintain muscular relaxation and bodily fluid stability; 7) The assessment protocol for skeletal muscle in both left and right lower limbs remains consistent.

#### AJ test

Before the jump test, participants completed a specific warm-up. This included 8 min of slow jogging at 8 km/h, and 7 min of dynamic stretching, The dynamic stretches included body weight squats, knee hugs, walking lunges, walking quadriceps stretches, high kicks, lateral lunges, and two maximum-intensity jump exercises. The protocol for the AJ test, whose reliability and validity have been substantiated in previous research (*ICC* = 0.97–0.99, *CV* = 2.1–2.8), was adhered to in this study (Sattler et al., 2012) and is as follows: 1) The examiner elucidated the procedure and prerequisites of the AJ test to the subjects and affixed six markers on the hip joint’s anatomical landmarks to ascertain the center of mass position. The apparatus was initiated once the subjects had acquainted themselves with the test motion and protocol; 2) In the AJ test, subjects were instructed to employ a 2- to 3-step approach of their preference and execute a jump augmented by an arm swing. They were to strive for maximal height in the ensuing rapid vertical jump, complemented by a vigorous arm swing. Subjects were guided to execute the jumping maneuver in a manner they deemed most suitable, emulating their offensive technique movement in volleyball matches or practice; 3) Each subject was to perform the AJ test thrice, interspersed with brief rest periods not exceeding 3 min; 4) Given that subjects were instructed to utilize their distinct movements to perform the AJ test, the specific procedure was relatively non-standardized. The primary objective of this study was to construct and validate the factors influencing AJ performance, necessitating acceptance of variations in individual movement patterns among the subjects.

#### General vertical jump test

General vertical jump assessments typically encompass CMJ, SJ, and DJ, each of which the participant performs thrice. During the CMJ, the participant initiates from a standing posture, promptly descends to a depth of their choosing, and subsequently propels upward as rapidly as possible, executing these movements in a fluid sequence. Throughout the assessment, the participant’s hands are consistently positioned at the hips. In the SJ, the participant individually adjusts to the initial jump position, maintains stillness for 2–3 s, and then vaults upward from this position as swiftly as possible. Similarly, the participant’s hands are maintained at the hips. The starting position for the jump is determined based on the natural position observed during the CMJ and is practiced before the initial SJ. At the outset of the DJ, the participant stands atop a 20 cm high platform, departs from it with one foot, and following a two-footed landing, promptly jumps upward. The participant is required to squat to the most natural position before the jump, maintaining a smooth jumping motion, but no further jumping instructions are given.

#### Data processing

All vertical jump tests leveraged the Qualisys high-speed video motion capture cameras. The accompanying Qualisys Track Manager system translated the analog signal into a primary digital signal, eliminated interference, and identified and marked the requisite points, capturing comprehensive AJ kinematic data. The data was modeled using Visual 3D software, which entailed importing the statically calibrated. C3D file, entering the athlete’s anthropometric data (height and weight), conducting pelvic modeling, and establishing a virtual center of mass point. This point was then applied to the dynamic data. C3D file to finalize the motion model. The kinematic data underwent processing via Butterworth second-order bidirectional low-pass filtering. Coding calculations were performed using the Pipeline program within the Visual 3D software, standardizing the kinematic data and exporting it as a. V3D file. Ultimately, the necessary processed data were imported into an Excel spreadsheet for final processing. The peak of the center of a mass displacement curve, post-processing with Visual 3D software, yielded the attack jump height.

After exporting three distinct muscle cross-sectional and longitudinal images of RF, VL, and LG procured from the LOGIQ 𝘦 NextGen Ultrasound device, the RadiAnt DICOM Viewer 2021.1.1 (64-bit) software facilitated the measurement of anatomical cross-sectional area, thickness, and pennation angle for all muscles. Muscle fascicle length was then estimated employing the formulas utilized by Fukunaga, Secomb, Albracht, and others (Formula 1) (Fukunaga et al., 1997; Secomb et al., 2015). For analytical purposes, the results from the left and right legs were amalgamated and averaged. All data were consolidated in an Excel spreadsheet for final processing. Furthermore, all ultrasound images were captured by a single technician to guarantee the reliability and validity of the data. The formulas are as follows:

$$FL=\frac{MT}{\sin PA}$$ (1)

#### Statistical analysis

The athletes’ basic physical information and key independent variables (including ACSA, MT, PA, and FL of the RF, VL, and LG), were entered into Microsoft Excel (Microsoft 365MSO, version 16.0.16425.20000, Microsoft Corporation™, Redmond, WA, United States) and imported into IBM SPSS 26.0 statistical software (SPSS Inc., Chicago, IL) for analysis. Descriptive statistics, including mean and standard deviation (Mean ± *SD*), were computed for each variable. The Shapiro-Wilk test was used to assess the variables’ normality (*p* ≥ 0.05). The inter-rater reliability between the B-mode ultrasound measurements of the participants was determined using the intra-class correlation coefficient (*ICC*). The within-subject variability of each test was assessed by calculating the coefficient of variation (*CV*). A paired *t*-test was used to analyze the differences between jump performance parameters.

A multiple linear regression model was built to determine the strongest predictor of jump height for elite male volleyball players. Pearson’s correlation coefficient (*r*_*p*_) or Spearman’s correlation coefficient (*r*_*s*_) was used to examine the relationship between the muscle structure variables of RF, VL, and LG and the AJ-Height variable, depending on the normality of the variables. After this analysis, the muscle structure variables of RF, VL, and LG that showed a strong correlation with the AJ-Height variable were selected and included in the multiple regression model to determine the key combination of variables that could predict AJ-Height. To ensure that the core variables were not eliminated due to the small sample size of this study, the standard for the inclusion of variables was set at *p* < 0.1 (Grant et al., 2019).

To ensure the reliability of our final model, we screened for confounding variables such as age, training years, weight, and height and included them in our multiple regression analysis using the same standards. This step not only increased the reliability and accuracy of the key predictor variables but also resulted in a new multiple linear regression model. During the multiple regression analysis, multicollinearity was assessed by eliminating variables with *VIF* > 5. The normality of the model was evaluated through visual inspection of *QQ* and residual plots, and further examination was conducted to assess the strength of acceptable models, explained by *Adjusted R* ^*2*^, and evaluated by ANOVA. To achieve good prediction accuracy, models were considered statistically significant at *p* ≤ 0.05, with an adjusted *R* ^*2*^ of 0.70 or higher, according to the sample size of the multiple linear regression analysis (Knofczynski and Mundfrom, 2008). The adjusted β values for the predictor variables and the level of residual standard error (*RSE*) in the model were obtained using IBM SPSS 26.0 statistical software, and the model was further examined using the bootstrap method (10,000 bootstrap samples).

## Result

### Examining variances among AJ, CMJ, SJ, and DJ, and the reliability of B-mode ultrasound imaging results

**FIGURE 3:**
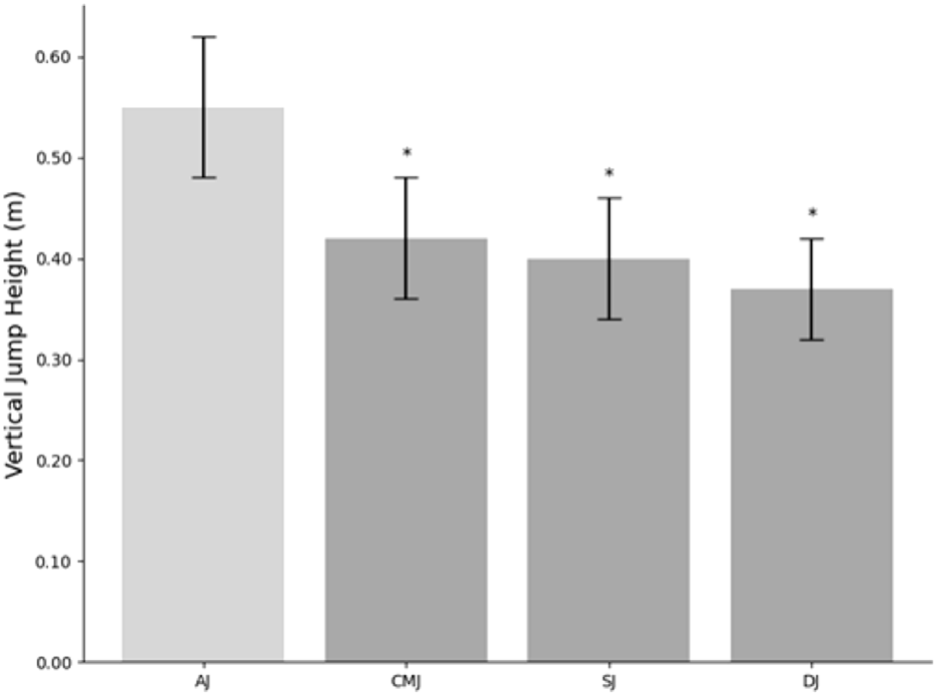
Mean jump height (n = 15) for Attack Jump (AJ), Countermovement Jump (CMJ), Squat Jump (SJ), and Drop Jump (DJ). *Represents a significant difference to attack jump performance. Values are reported as Mean ± *SD*.

According to the data displayed in Table 2; Figure 3, conspicuous and significant disparities are evident in the leap heights of AJ, CMJ, SJ, and DJ (*p* < 0.05). Concurrently, the information presented in Table 3 illustrates the substantial reliability of the B-mode ultrasound imaging tests conducted on the structure of the lower limb muscles (*CV* = 0.59–4.97%; *ICC* = 0.91–0.99).

**TABLE 3:** Muscle structure characteristics of RF, VL, and LG of elite male volleyball players (n = 15).

Variable		Unit	Mean ± *SD*	*ICC* (range)	*CV* (%)
RF	ACSA	cm^2^	14.19 ± 2.87	0.99 (0.99–0.99)	0.74
	MT	cm	2.50 ± 0.31	0.99 (0.98–0.99)	0.73
	PA	°	12.53 ± 3.28	0.94 (0.87–0.98)	3.37
	FL	cm	12.53 ± 3.36	0.97 (0.93–0.99)	3.22
VL	ACSA	cm^2^	36.17 ± 4.76	0.97 (0.93–0.99)	1.46
	MT	cm	2.83 ± 0.24	0.99 (0.97–0.99)	0.59
	PA	°	16.51 ± 2.96	0.95 (0.89–0.98)	3.00
	FL	cm	10.46 ± 2.28	0.91 (0.80–0.97)	3.75
LG	ACSA	cm^2^	8.03 ± 1.32	0.99 (0.97–0.99)	1.40
	MT	cm	1.27 ± 0.27	0.99 (0.97–0.99)	1.42
	PA	°	13.33 ± 3.22	0.92 (0.81–0.97)	4.59
	FL	cm	5.82 ± 1.62	0.94 (0.85–0.98)	4.97

ICC, intra-class correlation coefficient; CV, coefficient of variation; RF, rectus femoris; VL, vastus lateralis; LG, lateral gastrocnemius; ACSA, anatomical cross-sectional area; MT, muscle thickness; PA, pinnate angle; FL, fascicle length.

### Utilizing multivariate regression analysis to determine the predictive factors of lower limb muscle structure on the leap heights of AJ, CMJ, SJ, and DJ

Utilizing Pearson and Spearman correlation coefficients, robustly correlated lower limb muscular structure variables and confounding factors (e.g., Training Age) were incorporated into a multivariate linear regression model to construct an elite male volleyball player’s AJ-Height prediction model (*R* ^*2*^ = 0.73; *F* = 13.86; *p* < 0.001; *RSE* = 0.04) and conventional vertical jump height prediction models (CMJ, SJ, DJ), as detailed in Table 4. Concurrently, In our study, we employed the Bootstrap method to test results based on 10,000 bootstrap samples, determining bias values and errors, and calculating the Bca 95% confidence intervals (CI) to demonstrate the reliability of our model. Detailed results are presented in Table 5.

**TABLE 4:** Predictors of AJ-Height in elite male volleyball athletes based on multiple regression analysis (n = 15).

Dependent variable	Predictors	*β* (95% *CI*)	*Standardisedβ*	*t*	*Adjusted R* ^*2*^	*RSE* (m)	*F*	*p*
AJ-Height	Model				0.73	0.03	13.86	<0.001
	Training Age	−0.007 (−0.013, 0.001)	−0.35	−2.44	0.18			0.033
	VL-ACSA	0.01 (0.005, 0.014)	0.65	4.69	0.32			0.001
	LG-FL	0.02 (0.01, 0.030)	0.46	3.18	0.18			0.009
CMJ-Height	Model				0.51	0.04	8.41	0.005
	Training Age	−0.007 (−0.014, 0.001)	−0.41	3.42	0.11			0.046
	VL-ACSA	0.008 (0.003, 0.014)	0.64	−2.22	0.37			0.005
SJ-Height	Model				0.63	0.03	13.14	0.001
	Training age	−0.005 (−0.011, 0.001)	−0.36	−2.23	0.07			0.046
	VL-ACSA	0.009 (0.005, 0.013)	0.74	4.6	0.52			0.001
DJ-Height	Model				0.41	0.04	5.95	0.016
	Training Age	−0.006 (−0.012, 0.001)	−0.44	−2.16	0.14			0.052
	VL-ACSA	0.006 (0.001, 0.01)	0.55	2.67	0.25			0.021

AJ, attack jump; CMJ, countermovement jump; SJ, squat jump; DJ, drop jump; RSE, residual standard error; VL-ACSA, the anatomical cross-sectional area of vastus lateralis; LG-FL, fascicle length of the lateral gastrocnemius.

**TABLE 5:** Reliability of the multiple regression model assessed by the bootstrap method.

	Deviation value	Standard error	*Bca* 95% *CI*	
			Lower	Upper
Model	−0.29	0.45	1.15	1.64

*Bca* 95% *CI*, Bias-corrected and accelerated 95% confidence interval.

^a^ The bootstrap method was used to test the results based on 10,000 bootstrap samples.

## Discussion

This investigation aimed to elucidate the predictive capacity of RF, VL, and LG in their ACSA, MT, PA, and FL on the AJ performance of elite male volleyball players, distinguishing it from classic vertical jump types. Our study challenges the assumption of previous research that the relationship between muscle structure and performance in general vertical jumps (CMJ, SJ, DJ) can be directly extrapolated to the performance in AJ. Despite suggested correlations (Sattler et al., 2012), our data (Figure 3) suggest significant discrepancies exist, underscoring the unique characteristics of AJ in volleyball players. This may be due to the fact that different types of jumps possess specific jumping strategies and mechanical characteristics (Jidovtseff et al., 2014; Martinez, 2017; Reeser and Bahr, 2017), which could lead to varying influences of muscle structural features across different jump types. Therefore, conventional jumps may not fully represent volleyball-specific jump abilities, indicating possible limitations in using CMJ, SJ, and DJ as assessment tools for AJ performance. Our research findings also confirm that the muscle structures influencing the performance of the AJ differ from those involved in general vertical jumps. Consequently, greater attention should be devoted to understanding AJ performance and related muscular factors in volleyball athletes.

While the literature on the predictive role of lower limb muscular structures for AJ-Height is scant, making comparisons challenging, existing studies confirm the crucial role of leg muscles in general vertical jump performance. These findings underscore RF, VL, and LG as key muscles associated with lower limb functional capacity (Ruiz-Cardenas et al., 2018), likely due to their significant role in the lower limb kinetic chain. Notably, Our research has found that VL plays a key role in predicting jump height, and this result has been validated across different types of jumps. This may be due to the differences in the relative contribution rates of the four muscles of the quadriceps to knee extension torque. The current research results show discrepancies regarding the contributions of the quadriceps muscles (VL, VM, VI, RF) to knee joint extension torque (Leroux et al., 1997; Zhang et al., 2003; Narici et al., 2004; de Ruiter et al., 2008; Han et al., 2019; Lim et al., 2021). Based on recent studies, we can deduce the following relative contributions of the quadriceps muscles to knee joint extension torque: VL: 20.2%–61.4%, VM: 6.31%–23%, VI: 30%–51.8%, RF: 20.7%–28%. Interestingly, these studies have found that during submaximal isometric contractions at knee flexion angles of 60°/90°, VI and VM exhibit higher contribution rates. However, as the total knee extension torque increases, the relative contributions of VI and VM decrease, while those of VL and RF increase. This suggests that VL and RF may play a more significant role in high-intensity and explosive movements. Univariate regression analysis highlights VL-ACSA as the most significant variable in the AJ-Height prediction model, explaining 32% of the variance, aligning with previous reports on its significant correlation with general vertical jump performance and various lower limb activities (Methenitis et al., 2016; Agu-Udemba et al., 2018; Zaras et al., 2022). Observing muscle anatomical cross-sectional area is common in strength training research due to its pivotal link with maximal muscle force output (Franchi et al., 2018). The functional relevance of muscle anatomical cross-sectional area can be inferred from its contribution to overall strength (Morse et al., 2005). Hence, assessing the anatomical cross-sectional area of skeletal muscles is valuable. This study substantiates VL-ACSA as not only the lone muscular structural predictor for general vertical jump performance but also the optimal predictor within lower limb muscular structures for AJ performance.

The LG has been demonstrated to have a significant connection with lower limb functional capacity, one of the representative muscles in the calf group (Kumagai et al., 1999; Earp et al., 2010; Secomb et al., 2015; Coratella et al., 2019). In this study, LG-FL serves as the sole predictor for AJ-Height, unlike in general vertical jump performance. Univariate regression reveals LG-FL as the second most influential variable in the AJ-Height prediction model, accounting for 18% of its variance. Despite previous studies establishing a connection between LG muscle structure and general vertical jump ability, no correlation was identified between LG-FL and jump height (Earp et al., 2010; Secomb et al., 2015). Interestingly, a previous report on the relationship between lower limb muscle structure and sprint performance in 100 m sprinters found that faster sprinters typically have a longer LG-FL (Kumagai et al., 1999), consistent with our findings. This may be due to the specific demands of volleyball AJ skills on an athlete’s muscle groups. Our observations of the AJ movement pattern reveal that it primarily consists of a run-up, abrupt stop jump, and extensive arm swing motion. During the abrupt stop jump, a significant forward load is borne by the lower limbs, particularly the knee joint, to convert horizontal kinetic energy into vertical potential energy. With the forward shift of the knee joint and the continuous reduction of joint angles, the triceps surae gets progressively lengthened, storing substantial elastic potential energy. This possibly provides a degree of kinetic energy during the jump transition. However, most studies on vertical jumps (CMJ, SJ, DJ) employ standing jumps, often excluding arm swing motion. This might explain the divergence in research findings and how this unique “kinetic chain” could lead to noticeable differences in the load distribution of the lower limb “muscle chain.” It has been suggested that LG’s longer fascicle length allows for faster shortening speeds and results in a larger muscle mass (Kawakami et al., 1998). However, because fascicle length does not affect the physiological cross-sectional area, longer fascicle lengths might enhance AJ-Height through two mechanisms: first, longer fascicle lengths create higher maximum shortening speeds, leading to higher power output and an improved AJ-Height; second, strength development depends on the ability of a muscle to shorten at the required speed to generate sufficient force (Kearns et al., 1999). According to Hill’s equation (Hill, 1970), shortening speed increases as force decreases. Given the longer fascicle length of LG, it has a higher potential for maximum shortening speed, applying more significant force at a given shortening speed. The increased force translates into greater jump height, beneficial for AJ. This would explain why LG-FL can serve as one of the lower limb muscular structural predictors for AJ-Height. Furthermore, this could be due to our selection of elite volleyball players who have fascicle lengths surpassing those of athletes in other sports or untrained individuals. This observation is confirmed by studies analyzing differences in lower limb muscular structures among athletes at different levels, indicating that elite athletes have significantly longer fascicle lengths than non-elite athletes or untrained controls (Sarto et al., 2021; Zaras et al., 2022). This could be an adaptive response to long-term training and thus influenced by different sports and performance levels (Charles et al., 1998).

In summary, VL-ACSA and LG-FL are the best predictors of AJ-Height, while only VL-ACSA is a predictor of general vertical jump performance. This suggests a distinct difference in muscular structure predictors between AJ and other vertical jumps. As shown in Table 4, the standardized β coefficients for VL-ACSA and LG-FL in the AJ-Height model are 0.65 and 0.46, respectively. However, in the models for CMJ, SJ, and DJ-Height, the standardized β coefficient for VL-ACSA is 0.64, 0.74, and 0.55 respectively, with no predictive role shown for LG-FL. To assess the reliability of the AJ-Height model, we used the bootstrap method for testing, obtaining bias-corrected and Bca 95% *CI* based on 10,000 samples. According to Table 5, the AJ-Height model has a bias value of −0.29, a standard error of 0.45, and a Bca 95% *CI* of 1.15–1.64. These results indicate that the AJ-Height model has good reliability and stability, providing strong support for our discussion of its differences from other vertical jumps in terms of muscular structure predictors. Therefore, we can trust the predictive effect of the AJ-Height model in actual training and research. Moreover, we found a negative correlation with the number of years of training in all models. As shown in Table 4, the standardized β coefficient for the number of years of training in the AJ-Height model is −0.35, while in the models for CMJ, SJ, and DJ-Height, this coefficient is −0.41, −0.36, and −0.44 respectively. These data suggest that during AJ execution, the load distribution of lower limb muscular structure is different from that in general vertical jumps, especially in terms of the two predictors VL-ACSA and LG-FL.

As the only bipennate muscle in the quadriceps, the RF has significantly different anatomical characteristics from the other three muscles (Palastanga N, 2006). However, despite the substantial contribution of RF to knee extension torque (24%) (Narici et al., 2004), our study did not find any significant association between any muscular structure of RF and the heights of the four types of jumps. This may suggest that the contribution of the rectus femoris to vertical jump height is relatively low. This could be because the torques at the knee and hip are approximately equal, and the joint displacements of the knee and hip are very similar during the concentric force generation process, so the rectus femoris maintains the same length, making it almost unable to do any mechanical work on the skeleton. Moreover, compared to other lower limb muscles, the rectus femoris performs less work per unit volume. Therefore, although RF makes a large contribution to knee extension torque, its impact on vertical jump height is not significant due to the characteristics of the vertical jump movement (Wong et al., 2016). We also found that with an increase in training years, a negative correlation is observed with AJ-Height in elite male volleyball players. This could be influenced by factors such as aging, sports injuries, and the negative effects of many years of training. However, this does not mean that a longer training period will directly lead to a decrease in jump height. It should be clarified that this study controlled training years as a confounding variable, and it was not the main focus of the study. Future research can explore in more depth the mechanisms of how training years and related factors influence vertical jumping ability.

The results of this study reveal that lower limb muscle structure predictors play an important role in evaluating and improving the AJ ability of volleyball players. Coaches and athletes, to enhance performance in games, should focus on indicators such as VL-ACSA and LG-FL, and formulate corresponding training plans based on these indicators. Anthony J. Blazevich’s review (A. J. Blazevich, 2006) and María Ramírez-delaCruz’s systematic review with meta-analysis (Ramírez-delaCruz et al., 2022) confirm that long-term physical exercise can alter human muscle structure. These studies have found that resistance training and plyometric training not only improve muscle strength but also modify muscle structure, including ACSA, MT, PA, and FL. They also demonstrate that these training methods increase the ACSA, MT, PA, and FL of specific muscles. Intervention studies by M. V. NARICI (Narici et al., 1996) further support these findings, showing a significant increase in the cross-sectional area of the quadriceps, particularly the vastus lateralis (VL), in subjects who underwent 6 months of seated knee joint resistance training. Additionally, electromyography monitoring reveals that VL exhibits higher activation levels compared to other muscles during concentric and eccentric phases. Existing research has already shown that lower limb movements such as step-ups, forward lunges, and single-leg squats can effectively stimulate the VL, especially step-ups which surpass squats, lunges, deadlifts, etc., in terms of the level of VL activation (Ebben et al., 2009; Muyor et al., 2020). In addition, some research has found that front squats stimulate the vastus lateralis more than back squats (Contreras et al., 2016). Although there are currently no studies reporting which training methods have the greatest impact on LG-FL, there is research showing that after 20 men underwent eccentric training of the knee extensors, muscle fascicle length increased by 17%–19% in just 4 weeks, with pennation angle remaining unchanged. Therefore, it is speculated that eccentric training of the triceps surae might increase LG-FL (Baroni et al., 2013). As LG-FL is one of the effective predictors of AJ-Height in elite volleyball players and is different from general vertical jump performance, improving LG-FL might be one of the key factors in enhancing AJ performance. In conclusion, based on the muscular structure characteristics of athletes, specific training plans can be designed to strengthen VL-ACSA and LG-FL through the aforementioned movements and training methods. Furthermore, given that the AJ involves a stop-jump with a split-leg stance movement pattern, which differs from the typical bilateral jumping strategy, this may lead to asymmetrical lower limb muscle strength among volleyball players. Such asymmetry could negatively impact jump performance and increase the risk of injury. Consequently, training programs should account for bilateral differences and incorporate symmetry training to mitigate injury risk and enhance jump performance (Gao, 2022). These could not only potentially improve athletic performance, but also effectively reduce the risk of fatigue and injury to these muscle groups during training or competition. Therefore, designing targeted training plans to strengthen key muscular indicators is an important approach to improving the specialized jumping ability of volleyball players.

Our study has certain limitations. Firstly, the relatively small sample size may limit the robustness and generalizability of our results. Secondly, considering the muscle structural differences between men and women, our study focused only on elite male volleyball players. Hence, further research is needed to evaluate its impact on elite female volleyball players. Additionally, our study only involved the vastus lateralis, rectus femoris, and lateral gastrocnemius in the lower extremity kinetic chain, without including all lower extremity muscle groups. Lastly, our study only conducted a cross-sectional analysis of the correlation between muscle structure and jump height, lacking a comprehensive analysis of dynamics and kinematics. Overall, while our study provides a novel perspective and practical guidelines, the application of its findings should be approached with caution, requiring further research to verify and refine these findings.

## Conclusion

The findings of this study reveal a positive correlation between a larger VL-ACSA and vertical jumping performance. Moreover, LG-FL demonstrates a favorable association with the attack jump height of elite volleyball players, underscoring the significance of targeted training. It is advisable for those tasked with designing and implementing training programs to integrate exercises such as step-ups, forward lunges, single-leg squats, and eccentric strength training for the calf muscles. This holistic approach aims to optimize the specialized jumping performance of elite volleyball players. Furthermore, Future research could further investigate and validate the factors influencing specific jump (AJ) performance through the following approaches: increasing sample size and participant diversity, incorporating additional relevant muscle groups and their interactions with tendons, integrating dynamic and kinematic analyses, impact of lower limb asymmetry, conducting longitudinal studies, controlling for more confounding factors, and validating proposed interventions through experimental research.

## Data Availability

The raw data supporting the conclusions of this article will be made available by the authors, without undue reservation.
